# Distrust in the Health Care System and Adherence to Direct-Acting Antiviral Therapy among People with Hepatitis *C Virus* Who Inject Drugs

**DOI:** 10.3390/v16081304

**Published:** 2024-08-16

**Authors:** Akhila Padi, Irene Pericot-Valverde, Moonseong Heo, James Edward Dotherow, Jiajing Niu, Madhuri Martin, Brianna L. Norton, Matthew J. Akiyama, Julia H. Arnsten, Alain H. Litwin

**Affiliations:** 1 Department of Medicine, Washington University School of Medicine, St. Louis, MO 63110, USA; padi@wustl.edu; 2 Department of Psychology, Clemson University, Clemson, SC 29634, USA; iperico@clemson.edu; 3 Addiction Medicine Center, Department of Medicine, Prisma Health, Greenville, SC 29605, USA; 4 Department of Public Health Science, Clemson University, Clemson, SC 29634, USA; mheo@clemson.edu (M.H.); jdother@clemson.edu (J.E.D.); 5 School of Mathematical and Statistical Sciences, Clemson University, Clemson, SC 29634, USA; jiajinn@g.clemson.edu; 6 Department of Medicine, University of South Carolina School of Medicine, Greenville, SC 29615, USA; madhurim@email.sc.edu; 7 Albert Einstein College of Medicine, Montefiore Medical Center, Bronx, NY 10461, USA; brianna.norton@health.ny.gov (B.L.N.); julia.arnsten@einsteinmed.edu (J.H.A.); 8 Department of Medicine, Prisma Health, 605 Grove Road, Suite 205, Greenville, SC 29605, USA

**Keywords:** hepatitis C, PWIDs, DAAs, OAT

## Abstract

This study is a secondary analysis of a randomized clinical trial (October 2013–April 2017) involving 150 People Who Inject Drugs (PWIDs) with hepatitis *C virus* (HCV) seen in opioid agonist treatment programs in the Bronx, New York, and investigates the impact of distrust in the healthcare system on adherence to Direct-Acting Antivirals (DAAs) HCV treatment therapy among PWIDs. The distrust was scaled on a 9-item instrument and the adherence to DAA medications was measured using electronic blister packs. This study demonstrated a significant inverse relationship between levels of distrust and medication adherence: 71.8 ± 2.2% (se) vs. 77.9 ± 1.8%, *p* = 0.024 between participants with higher and lower distrust levels. Despite the absence of significant association of distrust with sociodemographic or substance use characteristics, these findings suggest that building trust within the healthcare system is paramount for improving adherence to DAAs among PWIDs. The results call for a healthcare approach that emphasizes trust-building through patient-centered care, sensitivity training, peer support, and health system reform to effectively address the treatment needs of this marginalized population.

## 1. Introduction

People who inject drugs (PWIDs) are a key population affected by the hepatitis C virus (HCV) epidemic. Globally, an estimated 14.8 million people inject drugs, of which 39% are at an increased risk of morbidity and mortality due to a HCV infection [1,2]. Despite accessible and highly effective HCV medication, treatment uptake remains alarmingly low among PWIDs—less than 20%—due to adherence concerns, ongoing drug use, psychosocial complexities, and the heightened reinfection risk associated with continuous drug use [3,4,5,6]. With the advent of direct-acting antivirals (DAAs) leading to sustained virologic response (SVR) rates exceeding 95%, elucidating barriers to HCV treatment adherence within this key demographic is imperative [7,8].

Despite the efficacy of DAAs, barriers to treatment uptake and adherence persist. Social barriers, such as health system access and navigation, housing, and criminalization are known barriers to treatment adherence [9,10,11]. Likewise, personal experiences and levels of trust are drivers of HCV treatment uptake and adherence [10,12].

Patient trust, cultivated through historical encounters and personal circumstances, is crucial in fostering engagement with healthcare services [13,14,15,16]. Trust is particularly influential in care initiation, adherence to medical care, enhancing service accessibility, and maintaining a high-quality patient–physician relationship, especially among vulnerable groups [16,17,18,19,20]. Mistrust in the healthcare system has been widely documented and linked to poor health outcomes, infrequent use of preventive care, and overall lower healthcare service utilization [21,22,23].

For PWIDs, diminished trust levels stem from stigmatization by healthcare workers, which can proliferate within communities through shared negative experiences. [17,19,24]. Stigma can manifest in different ways, each building deeper levels of distrust in the healthcare system. Discrimination and dismissive attitudes by providers are commonly felt among PWIDs, who report noticeable changes in attitudes, language, and levels of respect [25].

In addition to stigma, PWIDs have little trust in medicine or the healthcare system. Mistrust in the healthcare system is attributed to a number of factors, including perceptions of the quality of care related to health center size and the distrust in pharmaceuticals [26,27]. Trust in medicine has been eroded among the PWIDs community who report receiving mixed information regarding the spread and treatment of HCV [11,26,27].

Risk behaviors in PWIDs not only increase their vulnerability to HCV, but also to HIV and other health complications. Unfortunately, their engagement with primary healthcare is often limited to acute illnesses, which may result in delayed access to care and suboptimal use of preventive services, particularly evident in PWIDs living with HIV [24]. Such patterns of healthcare interaction have been associated with poor adherence to antiretroviral therapy, pivotal in reducing HIV transmissibility [28,29,30].

Due to the importance of adherence to DAAs in reducing the spread and morbidity of HCV, this study seeks to dissect how distrust in the healthcare system influences PWIDs’ adherence to treatment regimens. By examining the associations between distrust and adherence to DAAs among PWIDs, we aim to suggest potential modifications to HCV care models to better accommodate and address issues of distrust.

## 2. Materials and Methods

### 2.1. Study Design and Participants

This was a secondary analysis of a randomized clinical trial examining the effectiveness of three models of HCV care for PWIDs in opioid agonist treatment (OAT) programs: self-administered individual treatment (SIT), group treatment (GT), and modified directly observed therapy (mDOT) (clinicaltrials.gov NCT01857245) [31,32]. All 3 models of care included co-located, onsite care at the OAT program, which consisted of HCV care and substance use treatment. Enrollment began in October 2013 and participants were followed until April 2017. A total of 150 PWIDs with HCV were recruited from three OAT programs in the Bronx, New York. Eligible participants had to be ≥18 years of age, infected with HCV genotype 1, English or Spanish speaking, treatment naïve (or treatment experienced after 12/3/14), willing to receive HCV treatment onsite, and receiving methadone or buprenorphine at the medication window at least three times per week.

### 2.2. Medications

Participants received the following HCV medications in 7-day electronic blister packs: telaprevir, pegylated interferon, and ribavirin (TVR/IFN/RBV); sofosbuvir, pegylated interferon, and ribavirin (SOF/IFN/RBV); sofosbuvir and ribavirin (SOF/RBV); or a combination DAA regimen of sofosbuvir and simeprevir (SOF/SMV) or sofosbuvir/ledipasvir (SOF/LDV). Participants received different medication regimens based on clinical guidelines at the time they started treatment. Research visits were conducted at baseline, every 4 weeks during the first 12 weeks of HCV treatment (treatment week 4, 8, and 12), at the end of treatment (if the treatment regimen was ˃12 weeks, and 4, 12, and 24 weeks after treatment completion (follow-ups 4, 12, and 24). Detailed descriptions of the trial and study protocol are reported elsewhere [31].

### 2.3. Measures

At baseline, participants answered a series of questionnaires using audio computer-based self-interview technology (ACASI). The medical chart of each participant was systematically reviewed to extract information related to HCV-related outcomes and HIV coinfection.

#### 2.3.1. Sociodemographics

Sociodemographic characteristics were all collected at baseline and included age in years (median split: 52 or older and younger than 52), sex at birth (male and female), race/ethnicity (Black, Hispanic/Latino, White, and other), education level (lower than high school and higher or equal to high school), marital status (married/cohabitation and other), current health insurance status (yes and no), monthly income (median split: ≥USD 840 and <USD 840), homelessness (yes and no), employment status (employed, unemployed, retired, disabled, and other), and experience of living or time spent in a controlled environment such as jail and treatment programs (yes and no).

#### 2.3.2. Substance Use-Related Characteristics

Self-reported drug and alcohol use was assessed at baseline and every 4 weeks through the Addiction Severity Index-Lite version (ASI-Lite) instrument. In the present study, we used only a baseline substance use scale for number of days of use in the past 30 days. Specifically, this instrument was used to measure any use (yes for >0 days and no for 0 days) of alcohol or alcohol use for intoxication, as well as use of heroin, methadone, other opiates/analgesics, barbiturates, sedatives/hypnotics/tranquilizers, cocaine, amphetamines, cannabis, hallucinogens, and inhalants.

#### 2.3.3. Health-Related Variables

Health-related variables included baseline measures of perceived health status, depressive symptoms, and HIV co-infection. Perceived general health status was rated with a single-item 5-point Likert scale from excellent to poor based on the following statement: “In general, would you say your health is...”. We dichotomized the answers into two categories: Excellent/Very Good/Good and Fair/Poor. Depressive symptoms were measured using the Beck Depression Inventory-II (BDI-II), a 21-item self-reported questionnaire assessing the presence and severity of depressive symptoms that examines the severity of depression. The overall score ranges from 0 to 63 with greater scores representing greater severity, and we dichotomized into two groups: <17 and ≥17 based on a median split. Psychiatric illness (any major depressive disorder, psychotic disorder, general anxiety disorder, or current manic episode) was determined based on a medical chart review.

#### 2.3.4. Distrust Measures

Distrust in the healthcare system was measured at baseline using a 7-item scale developed by Altice and colleagues [19]. The distrust scale involved statements related to perceived distrust of hospitals, scientists, the pharmaceutical industry, and the government. Participants were asked to rate their agreement to each statement using a 5-point Likert scale from (1) strongly disagree to (5) strongly agree, with higher scores representing higher levels of distrust in healthcare system. In addition, 2 items were added to measure distrust to the healthcare system related to their HCV status with the same Likert scale scores. The Cronbach alpha of the 9-item instrument, with total score ranging from 9 to 45 with higher scores representing higher distrust levels, was estimated as 0.87 (95%CI: 0.82–0.90), which reflects acceptable internal consistency for the overall composite summary score. A summary distrust score was calculated for each participant as the average of each of the 9-item scores. The summary distrust score could range between one and five, and was dichotomized into higher levels of distrust (scores > 3) vs. lower levels of distrust (scores ≤ 3). We determined the threshold following that which was used for a previous paper using the same scale to reflect definite distrust with agree or strongly agree responses on average [33].

#### 2.3.5. Adherence Measures

Adherence to HCV medication was measured throughout the treatment period using electronic blister packs (Information Mediary Corporation, Ottawa, ON, Canada), which had an integrated sensor that recorded the exact time and date that each dose was removed. Adherence was defined as a continuous outcome calculated by using daily time frame (DTF) adherence, which was determined based on whether a blister was opened within a prescribed day. If opened, the DTF adherence was scored 1, and 0 otherwise. The two-week block adherence level was computed as the number of days with DTF adherence over the 14 days for longitudinal analysis; that is, a fraction of days out of 14 or the percent adherent days over two weeks, ranging from 0 to 100 in percentage points. Therefore, each participant contributed six bi-weekly adherence levels unless any or entire blister packs were unreturned. In addition, cross-sectional overall adherence level was computed as the average of the six two-week adherence levels for each participant to test if distrust is associated with an adequate adherence level of 70% or greater. This threshold was based on a PREVAIL study finding that the SVR rate was 90% or higher even among those who had <70% overall adherence [34]. Of note, no blister packs from N = 3 participants were returned and thus they were excluded from the adherence analysis.

### 2.4. Statistical Analysis

We used descriptive statistics to assess participants’ baseline characteristics and provide data on the distrust questionnaires. To identify baseline correlates of distrust measures, we compared, using chi-square/Fisher-exact tests and *t*-tests, sociodemographic, and drug use variables between those with summary scores ˃ 3 and those with summary scores ≤ 3. To compare the longitudinal bi-weekly measurements of the adherence levels between higher and lower distrust levels (summary scores ˃ 3 vs. ≤3), we applied a mixed-effects linear model with a first-order autoregressive covariance structure (to account for potential correlations of longitudinal adherence levels within individual patients), adjusting for time, time-by-distrust interaction, the study arm, psychiatric illness, and alcohol intoxication, all of which except the interaction were significantly associated with adherence levels in the PREVAIL study [32]. This analysis was conducted using only observed data assuming that missing bi-weekly adherence levels (<4%) due to unreturned blister packs during corresponding weeks were missing at random. In this modeling, all independent variables were considered discrete/categorical as opposed to continuous. In particular, the discrete time variable was coded with five dummy-coded binary variables representing the six bi-weekly time points. An unadjusted estimate of the distrust effects was obtained from a mixed-effects linear model with no adjusted variables. The estimated effect of primary interest obtained from the above models will quantify the percentage difference in adherence levels averaged over the entire treatment period between participants with higher vs. lower distrust levels. In addition, we compared the adequacy of adherence levels (i.e., overall adherence level 70% or greater) between participants with higher and lower distrust levels using a chi-square test. Statistical significance was declared if a two-sided *p* value was <0.05. Statistical analyses were conducted using SAS v9.4 and R v3.6.3.

## 3. Results

Most participants were male (64.7%), Hispanic (56.0%), and were ≥52 years old (53.3%). In total, 42,7% of participants had less than high school education and 48.7% were unemployed (Table 1). In the past 30 days, 18.7% of participants reported using heroin, 24% using cocaine, and 22% using other opiates or analgesics (Table 2). In addition, 14% were co-infected with HIV.

### 3.1. Associations between Participants’ Baseline Characterisitics and Overall Distrust Score

None of the baseline participants’ characteristics, including sociodemographic characteristics or substance use, were associated with overall composite distrust in the health system. However, 40% of participants reported higher levels of distrust in the healthcare system (summary scores ˃ 3). See Table 1 for sociodemographic factors and distrust and Table 2 for substance use and distrust.

### 3.2. Levels of Individual Distrust Scores

Table 3 shows participants’ responses to each item in the questionnaires assessing distrust in the healthcare system. The mean score for distrust in the healthcare system was 2.91 (SD = 0.77). However, more participants expressed higher distrust (item score >3) in the origins, knowledge, and handling of HIV by the government and scientist, as well as distrust in pharmaceutical companies. Specifically, almost half of the participants agreed or strongly agreed with the following statements: “I think that drug companies don’t tell me all the bad things that can happen with their medication” (45.3% with item score >3), “despite what the government would have you believe, I think that HIV was made in a laboratory” (43.3%), and “Medical scientists know more about HIV than they’re letting on” (44.0%).

### 3.3. Distrust and Adherence

**Figure 1:**
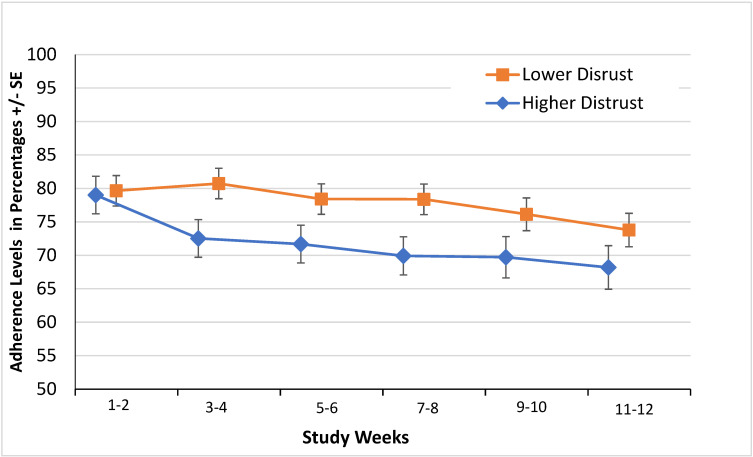
Estimated adjusted adherence levels among all patients (N = 147) between with higher and lower distrust of healthcare system based on mixed-effects linear model fitting. Note: No blister packs from N = 3 participants were returned for the analysis. The two-week block adherence level was computed as the number of days with DTF adherence over the 14 days, that is, a fraction of days out of 14 that were adherent, thus ranging from 0 to 100 in percentages. The bands above and below the estimated adjusted adherence levels represent the magnitude of standard error.

The linear mixed effects model showed that higher levels of distrust in the healthcare system were associated with lower adherence to medication (Figure 1). Participants with a summary score > 3 showed lower adjusted adherence levels than those with a summary score ≤ 3 (71.8 ± 2.2% (se) vs. 77.9 ± 1.8%, *p* = 0.024, diff = −6.0% (2.6%) 95%CI = (−11.2%, −8.0%)). That is, based on the estimates from the applied mixed-effects linear model, compared to participants who expressed lower distrust, those who expressed higher distrust in the health system had 6.0% lower adherence level in percentages, or 6.0% fewer adherent days (approximately 5 days out of the total 12-week period), during the treatment period. Detailed results from adjusted and unadjusted estimates are presented in Table 4. In addition, patients with low distrust had significantly more adequate adherence levels (70% or greater) compared to those with high distrust level (80% [72/90] vs. 64.9% [37/57], *p* = 0.042, diff = 15.1% 95%CI = (0.2%, 30%)).

## 4. Discussion

To our knowledge, this is the first quantitative study to examine the association between distrust in the healthcare system and adherence to DAA treatment adherence among PWIDs with HCV. The findings highlight a significant association between higher levels of distrust and lower adherence to DAAs, which underscores the necessity of addressing trust-building in HCV treatment models at the system level. It is imperative to consider the complex interplay of factors contributing to such distrust, which could include historical prejudices, systemic biases, and personal experiences with stigmatization. The absence of a correlation between participants’ baseline sociodemographic characteristics and substance use history with distrust levels suggests that distrust may be more deeply rooted in collective experiences within healthcare systems than has been previously understood, especially among marginalized populations such as our study population.

Our findings align with those of similar studies examining HCV treatment and adherence among PWIDs. Studies have shown PWIDs experience stigma and discrimination when accessing healthcare services, which can explain low treatment initiation and adherence [12,25]. Similarly, studies with homeless PWIDs, where HCV screening and treatment are low, have identified distrust in the healthcare system and health professionals as primary barriers to care [26,27].

The history of discrimination and institutionalized racism in healthcare has led to and perpetuated an attitude of distrust among minority and marginalized populations [35,36]. The result of perceived discrimination and distrust often leads to a poor retention in care and medication adherence, as patients question the efficacy and effects of medication [37,38]. Distrust in the healthcare system among minority groups and marginalized populations can reliably predict non-adherence to antiretroviral therapy (ART) [18,39,40].

The persistent stigmatization and discrimination faced by PWIDs contribute to a cyclical pattern of distrust, nonadherence, and further health disparities. Healthcare providers and systems must be cognizant of the power dynamics at play and strive for a more empathetic and inclusive approach. Integrating peer support and advocacy within treatment models could serve as a bridge to rebuild trust, as peer relationships are less likely to be affected by the power imbalances that characterize patient-provider interactions, and trust remains central in the medication initiation process [19].

Furthermore, the study emphasizes the need for personalized care strategies that address the unique challenges faced by PWIDs. By offering flexible treatment options and considering patient’s preferences and past experiences, healthcare systems can foster a more trusting and supportive environment. These strategies could potentially mitigate the impact of distrust on medication adherence. Our results resonate with existing literature that highlights the need for patient-centered care in improving health outcomes among marginalized populations [41].

The observed lower adherence levels among participants with higher distrust levels present an opportunity to reshape healthcare delivery, particularly with this vulnerable population. Regular training on sensitivity and bias for healthcare workers, implementation of non-stigmatizing policies, and increased accountability within the healthcare system are potential measures to enhance trust. Additionally, clear communication and transparency in treatment plans, particularly concerning the effectiveness and side effects of medication, could help mitigate some of the distrust [42].

The limitations of the study include its reliance on self-reported measures of drug use, which are subject to reporting biases. The relatively small sample size may have hindered our ability to detect associations between sociodemographic and drug-related factors and distrust. Additionally, the study’s focus on PWIDs within opioid agonist treatment programs may not be generalizable to all PWIDs populations. Future research should explore longitudinal assessments of distrust and adherence, as well as interventions designed to mitigate distrust in this population.

In conclusion, our study’s findings affirm the critical need for trust-building measures within the healthcare system to improve adherence to HCV treatment among PWID. While it is unclear whether interventions alone can improve trust in the healthcare system, screening patients with HCV with questions related to distrust in the healthcare system, prior to initiation of therapy may highlight individuals who are at risk of nonadherence to DAA therapy. By acknowledging and addressing the multifaceted origins of distrust, healthcare providers can tailor interventions to cultivate a treatment environment where PWIDs feel valued, understood, and engaged in their care, ultimately leading to better health outcomes and moving closer to achieving the World Health Organization (WHO) 2030 goals of eliminating HCV.

## Figures and Tables

**Table 1:** Participant characteristics at baseline and distrust in healthcare systems.

	Distrust in the Healthcare System * (N = 150)		
Characteristic at Baseline	Summary Score > 3 *n* = 60 (40.0%)	Summary Score ≤ 3 *n* = 90 (60.0%)	*p*
Study Arm **			0.239
mDOT	16 (31.4%)	35 (68.6%)	
GT	23 (47.9%)	25 (52.1%)	
SIT	21 (41.2%)	30 (58.8%)	
Age (median-split)			0.616
≥52 years old	30 (37.5%)	50 (62.5%)	
<52 years old	30 (42.9%)	40 (57.1%)	
Sex at birth			1
Male	39 (40.2%)	58 (59.8%)	
Female	21 (39.6%)	32 (60.4%)	
Race/Ethnicity			0.633
Black	16 (40.0%)	24 (60.0%)	
Hispanic/Latino	34 (40.5%)	50 (59.5%)	
White	3 (25.0%)	9 (75.0%)	
Other	7 (50.0%)	7 (50.0%)	
Education			0.098
Lower than High School	31 (48.4%)	33 (51.6%)	
≥High School graduate	29 (33.7%)	57 (66.3%)	
Marital status			0.663
Married/cohabitaion	29 (42.6%)	39 (57.4%)	
Other	31 (37.8%)	51 (62.2%)	
Current health insurance			1
Yes	56 (41.2%)	80 (58.8%)	
No	4 (40.0%)	6 (60.0%)	
Monthly income(median-split)			1
≥$840	30 (39.5%)	46 (60.5%)	
<$840	30 (40.5%)	44 (59.5%)	
Employment			0.489
Employed	2 (16.7%)	10 (83.3%)	
Unemployed	30 (41.1%)	43 (58.9%)	
Retired	7 (50.0%)	7 (50.0%)	
Disabled	18 (41.9%)	25 (58.1%)	
Other	3 (37.5%)	5 (62.5%)	
Homelessness			0.449
Yes	16 (47.1%)	18 (52.9%)	
No	44 (37.9%)	72 (62.1%)	
Experience of living in a controlled environment ***			1
Yes	10 (40.0%)	15 (60.0%)	
No	50 (40.0%)	75 (60.0%)	
Perceived general health status			0.077
Excellent/Very Good/Good	23 (31.9%)	49 (68.1%)	
Fair/Poor	37 (47.4%)	41 (52.6%)	
Depression			0.150
BDI-II **** < 17	28 (34.1%)	54 (65.9%)	
BDI-II ≥ 17	32 (47.1%)	36 (52.9%)	
Curret psychiatric illnesses			0.283
Yes	30 (44.8%)	37 (55.2%)	
No	30 (36.1%)	53 (63.9%)	
HIV co-infection			0.848
Yes	8 (38.1%)	13 (61.9%)	
No	52 (40.3%)	77 (59.7%)	

Note: The percents represent row percentages. * Scores > 3 represent higher distrust level and scores ≤ 3 represent lower trust level. ** mDOT: modified directly observed therapy; GT: group treatment; SIT: self-administered individual treatment. *** Experience of living or time spent in a controlled environment such as jail and treatment programs. **** BDI: Beck Depression Inventory.

**Table 2:** Past 30-day substance use (no day vs. any days) and distrust in healthcare systems.

	Distrust in Healthcare System * (N = 150)		
Drug Use in the Past 30 Days	Summary Score > 3 *n* = 60 (40.0%)	Summary Score ≤ 3 *n* = 90 (60.0%)	*p*
Heroin			0.764
Yes	10 (35.7%)	18 (64.3%)	
No	50 (41.0%)	72 (59.0%)	
Other Opiates/Analgesics			0.903
Yes	14 (42.4%)	19 (57.6%)	
No	46 (39.3%)	71 (60.7%)	
Cocaine			0.725
Yes	13 (36.1%)	23 (63.9%)	
No	47 (41.2%)	67 (58.8%)	
Alcohol			0.640
Yes	17 (36.2%)	30 (63.8%)	
No	43 (41.7%)	60 (58.3%)	
Alcohol Intoxication			0.969
Yes	15 (41.7%)	21 (58.3%)	
No	45 (39.5%)	69 (60.5%)	
Methadone **			1
Yes	59 (39.9%)	89 (60.1%)	1
No	1 (50.0%)	1 (50.0%)	
Barbiturates			
Yes	2 (33.3%)	4 (66.7%)	1
No	58 (40.3%)	86 (56.7%)	
Sedatives/Hypnotics/Tranquilizers			0.355
Yes	16 (48.5%)	17 (51.5%)	
No	44 (37.6%)	73 (62.4%)	
Amphetamines			0.150
Yes	0 (0.0%)	4 (100.0%)	
No	60 (41.1%)	86 (58.9%)	
Cannabis			0.288
Yes	21 (47.7%)	23 (52.3%)	
No	39 (36.8%)	67 (63.2%)	
Hallucinogens			1
Yes	2 (40.0%)	3 (60.0%)	
No	58 (40.0%)	87 (60.0%)	
Inhalants			0.400
Yes	1 (100.0%)	0 (0.0%)	
No	59 (39.6%)	90 (60.4%)	

Note: The percents represent row percentages. * Scores > 3 represent higher distrust level and scores ≤ 3 represent higher trust level. ** As the participants were recruited from opioid agonist treatment programs, methadone was detected in the context of dispensed treatments rather than illicit use.

**Table 3:** Distrust in healthcare system (N = 150).

Distrust in the Healthcare System Questionnaire Item (1 to 5; 5 Highest Distrust)	N (%) with Individual Item Scores > 3	Score M (SD)
I think that I have been experimented on in hospitals without being told	21 (14.0%)	2.23 (1.08)
I think that drug companies don’t tell me all the bad things that can happen with their medication	68 (45.3%)	3.11 (1.16)
I think there is a cure for AIDS, but the government is keeping it from us	57 (38.0%)	3.10 (1.16)
Despite what the government would have you believe, I think that HIV was made in a laboratory	65 (43.3%)	3.21 (1.11)
I suspect that my blood or urine is being tested for things I’m not told about	43 (28.7%)	2.81 (1.06)
Medical scientists know more about HIV than they’re letting on	66 (44.0%)	3.26 (1.05)
I think that hospitals are more concerned with making money than healing me	54 (36.0%)	2.93 (1.17)
Despite what the government would have you believe, I think that hepatitis C was made in a laboratory	22 (14.7%)	2.67 (1.01)
Medical scientists know more about hepatitis C than they’re letting on	39 (26.0%)	2.85 (1.11)
Overal Summary Score	60 (40.0%)	2.91 (0.77)

**Table 4:** Adjusted and unadjusted effects of distrust on bi-weekly longitudinal adherence levels.

	Unadjusted Estimates		Adjusted Estimates	
Effects	Estimates (95% CI)	*p*	Estimates (95% CI)	*p*
Overall Distrust (Higher vs. Lower) *	−5.9% (−11.2%, −0.7%)	0.027	−6.0% (−11.3%, −0.8%)	0.024
Time (Discrete)			***	0.030 ****
Distrust × Time Interaction			***	0.137 ****
Study Arm **				0.040 ****
mDOT vs. GT			4.1% (−1.9%, 10.1%)	0.179
mDOT vs. SIT			7.7% (1.8%, 13.7%)	0.011
GT vs. SIT			3.6% (−2.4%, 9.7%)	0.237
Alcohol Intoxication (Yes vs. No)			−7.1% (−12.8%, −1.4%)	0.015
Psychiatric Illnesses (Yes vs. No)			−4.4% (−9.3%, 0.6%)	0.082

Note: The percents represent adherence levels in percentages. * Difference in estimated adherence levels in percentages. ** mDOT: modified directly observed therapy, SIT: Self-administered Individual treatment; GT: group treatment. *** See Supplementary Table S1 for detailed contrasts between estimated differences in adherence levels across all combinations of time points and distrust levels, i.e., between pairs of different time points and between higher and lower distrust levels at different or same time points. These estimated contrasts are independent of choice of a reference time point for constructing dummy-coded binary variables. **** Based on Type III F-tests.

## Data Availability

The data used in this study are available upon reasonable request to the corresponding author.

## Supplementary Materials

The following supporting information can be downloaded at: https://www.mdpi.com/article/10.3390/v16081304/s1. Table S1: Contrasts between estimated differences in adherence levels across all combinations of time points and distrust levels.
